# Exposure to a Fungal Volatile Compound: Wålinder et al. Respond

**Published:** 2006-06

**Authors:** Robert Wålinder, Dan Norbäck, Gunilla Wieslander, Lena Ernstgård, Gunnar Johanson

**Affiliations:** Department of Medical Sciences/Occupational and Environmental Medicine, University Hospital, Uppsala, Sweden, E-mail: robert.walinder@medsci.uu.se; Division of Work Environment Toxicology, Institute of Environmental Medicine, Karolinska Institutet, Stockholm, Sweden

There are different opinions on the use of Bonferroni’s corrections. Everitt (1995) stated that it gives too conservative estimates if there are more than five tests performed. In environmental medicine, one exposure can have different health effects, so it is reasonable to test for different types of effects on different organs. We prefer to perform conventional statistical tests, without Bonferroni correction, and look at the pattern of significant effects and their biologic plausibility.

We did not perform 76 comparisons (Wålinder et al. 2005); we actually performed 25 tests on 13 physiologic variables based on differences before and after exposure. A fourteenth variable (vital staining) was tested only after exposure. Repeated measurement analysis was performed on blink frequency (60 consecutive measurements of 2 min each) and one questionnaire with 10 questions was administered at six different times. This is a total of 37 tests performed on 25 variables, having five significant values, of which one was highly significant (*p* < 0.001).

Moreover, all tests point in the same direction—mucosal effects of the exposure. We did not find the same effects over time for control exposure. There is, of course, the possibility that some of the significant effects were due to chance, and we were quite modest in our conclusions, using the words “may” or “might be.” Because our study is the first exposure-chamber study on 3-methylfuran (3-MF), more studies are needed to determine final conclusions.

Blink frequency was measured only during the 2 hr of exposure in the chamber. Therefore, there is no preexposure baseline data available at time 0. Eye effects occur quickly; a rapid effect of the exposure on blinking frequency can occur during the first 2 min of exposure to 3-MF inside the chamber, possibly followed by later adaptation (8.8 blinks/min during the first 2 min, compared with a mean of 7.6 blinks/min during the whole period of exposure).

It is true that there was a numerical increase in break-up time at exposure, which could be in agreement with increased blink frequency. The fatty layer on the tear film is produced by the glands of the eyelids. Therefore, an increased blinking frequency could produce more secretion from the meibomian glands and therefore a longer break-up time.

Regarding lung function, transient effects of environmental exposure (as well as diurnal variation, which we controlled for by performing the experiment at the same time) may affect either forced vital capacity (FVC) or forced expiratory volume in 1 sec (FEV_1_), or both. Physiologically and numerically, the decrease was of the same order, but statistically the outcome was different. The decreases were 0.1 L for FVC and 0.08 L for FEV_1_ after exposure to 3-MF. The magnitude of the effect was clinically small, but it was significant at group level for FVC. Small pulmonary effects may have large health effects in a population (Künzli et al. 2000.)
